# Quantitative Evaluation of Chinese Herb Medicine in the Treatment of Sialorrhea and Frequent Nighttime Urination in Patients with Parkinson's Disease

**DOI:** 10.1155/2017/3045260

**Published:** 2017-04-18

**Authors:** Chuanhe Sun, Dandan Wang, Wenfei Jiang, Weilong Liao, Penglin Gao, Weidong Pan, Te Liu

**Affiliations:** ^1^Department of Neurology, Shuguang Hospital Affiliated to Shanghai University of Traditional Chinese Medicine, Shanghai, China; ^2^School of Pharmacy, Shanghai University of Traditional Chinese Medicine, Shanghai, China; ^3^Shanghai Geriatric Institute of Chinese Medicine, Longhua Hospital, Shanghai University of Traditional Chinese Medicine, Shanghai, China

## Abstract

*Aims*. To evaluate the efficacy of Lian-Se formula (LSF), one Chinese herb formulation for treating sialorrhea and frequent overnight urination in patients with Parkinson's disease (PD).* Methods*. 96 PD patients suffering from sialorrhea and/or frequent nighttime urination were divided into two groups: an LSF group (*n* = 48) treated with LSF for 6 weeks and a placebo group (*n* = 48) treated with a placebo formula whose appearance and taste were the same as LSF for 6 weeks. All patients were treated by standard antiparkinsonism medicine according to the PD guideline of China. The changes of the quantity of saliva (QS) (mL), frequency of nighttime urination (FNU) and early sleep activity (ESA), and nocturnal activity (NA) by analyzing actigraphic records as the primary results and the total score of unified Parkinson's disease rating scale (UPDRS) and the Epworth Sleepiness Scale (ESS) as the secondary results were used to evaluate the clinical efficacy in both groups.* Results*. There were no significant differences in the baseline values of QS, FNU, NA, ESA, UPDRS total score, and ESS between the two groups. At the end of week 6, the QS, FNU, NA, and ESA in the LSF group showed superior results to those of the placebo group with no differences in the total UPDRS score between the two groups during the investigation. The ESS was significantly improved at the end of week 6 compared with the baseline and the placebo group. Laboratory test results indicated there were no side effects in either group.* Conclusion*. The findings of LSF treatment have clear clinical effects in patients with sialorrhea and frequent overnight urination. LSF thus appears to be a potential choice as an additional drug that can improve the sialorrhea and frequent overnight urination symptoms of PD patients.

## 1. Background

Parkinson's disease (PD), traditionally, was clinically defined as a fundamental motor disorder that included involuntary tremulous motion with lessened voluntary muscular power and abnormal postures [1]. Nonmotor symptoms (NMS) such as depression, pain, fatigue, bladder dysfunction (dysuria and frequent nighttime urination), sialorrhea, mood, constipation, and sleep disorders and even autonomic nerve disorders, cognitive decline, and delusions are clearly noted by neurological researchers from clinical investigations [2]. It is now evident that NMS occur not only across all motor stages of PD but also in premotor stages. The symptoms of frequent nighttime urination and sialorrhea may influence motor symptoms and lead to the development of other NMS such as insomnia, mood disturbances, and depression in PD patients and, sometimes, may disrupt the quality of life to a greater extent than motor disorders. Traditional Chinese medicines (TCM) ameliorate various symptoms, particularly aging-related symptoms [3], and hence are likely to be beneficial for chronic diseases such as PD. Both frequent nighttime urination and sialorrhea are explained in the theory of TCM in terms of dysfunction of body fluid control. TCM theory considers the symptoms to be a Qi deficiency in the spleen and kidneys. We used Lian-Se formula (LSF), a TCM herbal formulation, which can improve the Qi function of the body and has exhibited an inducing astringency function for treating patients with PD compared with placebo herbs. An actigraph, which is a quantitatively evaluative device [4], was used to estimate the effects of 6-week TCM treatment in patients.

## 2. Subjects and Methods

### 2.1. Subjects

A prospective double-blind controlled study with consecutive enrollment of 96 subjects affected by idiopathic PD was carried out in patients admitted to the Department of Neurology of Shuguang Hospital Affiliated to Shanghai University of Traditional Chinese Medicine (Table 1). The diagnosis of PD was established when two of the main symptoms (bradykinesia, tremor, rigidity, and postural reflex abnormality) were presented [5]. The patients were at least 40 years of age and were evaluated in the middle of their levodopa dose cycle at maximal mobility (“on”) for the severity of parkinsonism. For a patient to be included in the study, they must have had at a minimum sialorrhea (≧15 mL daily) and/or frequent overnight urination (≧3 times per night). Extrapyramidal syndromes due to another central nervous disease were excluded. If a patient suffered from other symptoms or diseases which may influence the quality of sleep or the quantitative evaluation of sialorrhea such as dementia, chronic obstructive pulmonary disease (COPD), obstructive sleep apnea hypopnea syndrome (OSAS), pain symptoms, alcohol or drug addiction, angina pectoris, stroke, bell palsy (facial paralysis), mumps, or restless leg syndrome, they were also excluded from the study. In addition, in order to evaluate the overall quality of sleep, outcome measures on quality of life were recorded using the Epworth Sleepiness Scale (ESS) [6]. The total score of the unified Parkinson's disease rating scale (UPDRS) was also used to evaluate fluctuations of the disease.

All patients underwent a neurologic examination and routine blood tests (including serum iron and ferritin, B12 vitamin, and folate concentrations). Patients with any abnormality in these tests or with an apnea hypopnea index greater than 5 were also excluded.

The study was approved by the Ethics Committee of Shuguang Hospital Affiliated to Shanghai University of TCM and was performed according to the principles outlined in the Declaration of Helsinki.

### 2.2. Chinese Herb Medicine and Placebo Treatment

All subjects were randomly subdivided into 2 subgroups: a treatment group (*n* = 48, Chinese herb medicine Lian-Se formula, LSF) and placebo group (*n* = 48, placebo Chinese medicine). The LSF granule contains 8 kinds of TCM herbs:* Diaphragma juglandis*,* Cistanche deserticola*,* Astragalus mongholicus*,* Schisandra chinensis*,* Rhizoma Atractylodis*,* Rosa laevigata*,* Radix Paeoniae Rubra*, and* corn stigma*. Placebo granules consisted of 5 kinds of herbs:* Largehead Atractylodes Rhizome*,* Poria cocos (Schw.) wolf*,* Jobstears seed Malt*, and* Chinese date *[7]. These 5 herbs have no activity in terms of TCM. Patients were instructed to take one package (8 g) of LSF or placebo granules three times a day at least 30 min before or after the ingestion of other drugs for 6 consecutive weeks. The shape and color of the LSF and placebo granules were very similar and could not be distinguished from one another by appearance or taste. LSF and the placebo granules were made by the manufacturing laboratory of Shuguang Hospital Affiliated to Shanghai University of TCM.

### 2.3. Subjective Evaluation of Sialorrhea and Overnight Urine Output

In order to determine the quantity of sialorrhea in the patients, the patients or their caregivers were trained by the nurses to estimate the quantity of saliva (QS) using towels, small cups, and tissue paper. The average of two days of sialorrhea was the primary outcome for evaluating the severity of sialorrhea. The frequency of nighttime urination (FNU) was counted to assess the changes in overnight urine output.

### 2.4. Nocturnal Actigraphic Recording

Nocturnal actigraph recording was carried out after an adaptation night in a standard sound-attenuated sleep room (at a ward in the Department of Neurology, Shuguang Hospital Affiliated to Shanghai University of TCM, or at home). Subjects were not allowed to consume caffeinated beverages beginning the afternoon preceding the recordings and were allowed to sleep until their spontaneous awakening in the morning. Lights-out time was based on each individual's habitual bedtime and ranged between 22:30 and 23:00. All subjects wore an actigraph, which is a small watch-type activity monitor equipped with a computer (*Micro*-*Mini*-*Motionlogger*, Ambulatory Monitoring, Inc., Ardsley, New York) [8], on the wrist of the affected dominant side or worse side for 2 consecutive days in the series time windows (before, at the end of week 2, and week 6 after taking TCM) during the observation period. Zero-crossing counts were recorded every minute by the actigraph to register and quantify the level of physical activity [9]. After recording, the data were transmitted to an external computer by software installed on the device. We plotted the activity scores for two consecutive days to determine the nocturnal activity of each patient. The data acquired from the actigraph during the observation period were separated into two time periods for analysis: early sleep activity (ESA, each first one hour of night bedtime activity) and the nocturnal activity (NA, all nocturnal bedtime activity) [10].

The primary outcome was the change in quantity of saliva (QS), frequency of nighttime urination (FNU), and the activity level of NA and ESA of the patients. The secondary outcome was the changes in the UPDRS score and ESS score. The outcome measures were assessed before (baseline), at the end of week 2, and at the end of week 6 during the 6 weeks' observation period.

## 3. Statistical Analysis

Repeated-measure ANOVA was conducted to test the differences among changes in outcomes at baseline and at the end of week 2 and week 6. When a significant difference was detected, a post hoc test (Bonferroni test) was conducted between the LSF and placebo groups in order to compare the levels of QS, FNU, NA, and ESA and the UPDRS and ESS scores. Differences at baseline between the LSF and placebo groups were analyzed using the *t*-test. A significant difference was defined as *p* < 0.05. SPSS windows version 17.0 was used for statistical analyses. All data are expressed as the mean ± standard deviation.

## 4. Results

At the end of week 6, 4 patients in the LSF group (dependency 91.66%) withdrew from the study; two patients were afraid of excessive water intake when ingesting the LSF granules and another two patients withdrew from the trial without any reason. Five patients in the placebo group (dependency 89.58%) withdrew from the study. Three patients discontinued because they refused to take more water, and others withdrew from the trial due to a lack of effects. No significant differences were found in the baseline values of QS, FNU, NA, and ESA and UPDRS total score and ESS between the two groups (Table 1).

**Figure 1:**
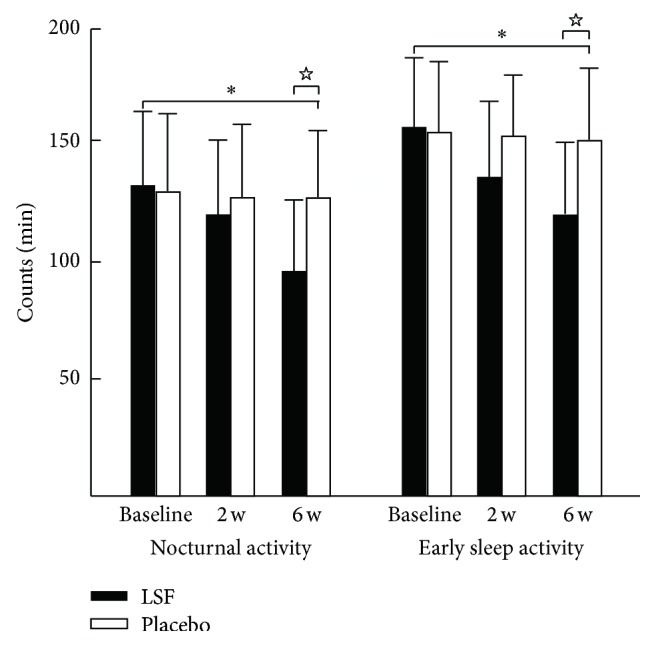
Illustrations demonstrating the fluctuation of nocturnal activity (left side) and the early sleep activity (right side) of patients with Parkinson's disease before (baseline) and week 2 and week 6 after the traditional Chinese medicine (TCM) treatments. The histograms with error bars between the illustrations show the mean activities for each subject as the arrows indicate. ^*∗*^*p* < 0.05, compared with before treatment for the Lian-Se formula (LSF) group, ^☆^*p* < 0.05, compared with the placebo group.

**Figure 2:**
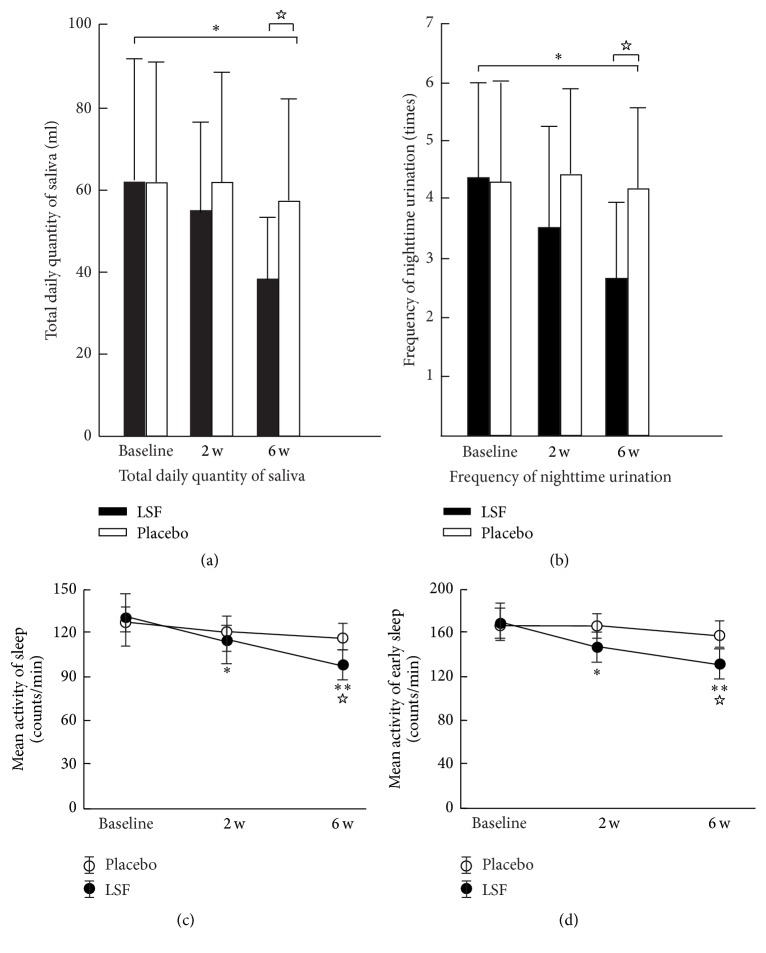
Changes in quantity of saliva (a) and the frequency of nighttime urination (b) before and after the TCM treatment. (c) and (d) present the changes in mean activity of sleep (c) and the mean activity of early sleep (d) in the two groups of patients. ^*∗*^*p* < 0.05 and ^*∗∗*^*p* < 0.01 compared with before treatment for Lian-Se formula group; ^☆^*p* < 0.05, compared with placebo group.

**Figure 3:**
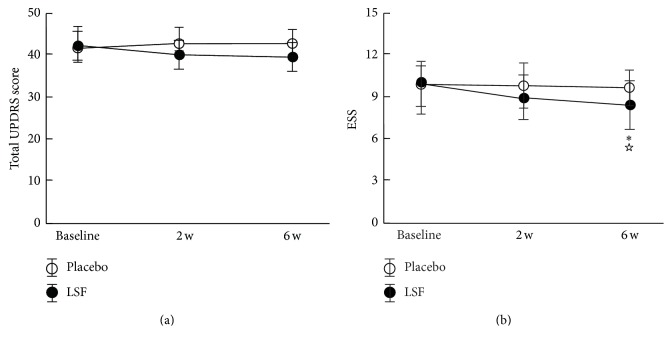
Changes in the total score of the unified Parkinson's disease rating scale (UPDRS) (a) and Epworth Sleepiness Scale (ESS) (b) before and after the treatments. ^*∗*^*p* < 0.05, compared with before treatment for the Lian-Se formula (LSF) group; ^☆^*p* < 0.05, compared with the placebo group.

Examples of the actigraphic recordings for the two groups illustrated the fluctuations in nighttime activity for the two groups (Figure 1). Reduced nighttime activity after 6 weeks treatment was observed in the LSF group compared with before. A similar difference between before and after the treatment was not observed in the placebo group. The QS, FNU, NA, and ESA values in the LSF group were superior to those in the placebo group at the end of week 6 (Figure 2). The total score of the UPDRS indicated there were no such differences between the two groups during the investigation (Figure 3(a)). The ESS was 7.33 ± 2.46 in the LSF and 10.51 ± 2.52 in the placebo group (Figure 3(b)), and the difference was significant (*p* < 0.044) at the end of week 6.

There were no abnormal laboratory test result values in either group.

## 5. Discussion

The Chinese physician Dr. TU Youyou from the China Academy of Chinese Medical Sciences was awarded the Nobel Prize for Medicine and Physiology in 2015, the highest award for physicians, for her excellent work on sweet wormwood (Artemisia annua) in malaria research [11], and as a result TCM has been attracting more attention around the world in the field of medicine. More and more integrative researchers are hoping to identify innovative treatments for difficult diseases that cannot be improved or treated by Western medicine. Sialorrhea and frequent nighttime urination are two very painful NMS for PD patients and there is a lack of effective treatments in Western medicine. The treatment of PD requires integrative medicine to assist Western medicine as a complementary and alternative medicine (CAM) to improve the motor and nonmotor symptoms of PD patients [12]. In this study, we demonstrate that LSF, a TCM, ameliorates these two nonmotor symptoms using evaluation methods that included QS, FNU, NA, and ESA parameters together with conventional ESS and UPDRS scores. LSF did not induce any significant adverse effects and was tolerable by more than 90% of the participants.

We previously demonstrated that the use of TCM could improve some motor and nonmotor symptoms of patients with PD [7, 13]. According to TCM theory, sialorrhea and frequent nighttime urination are problems related to body fluid control, which is controlled by Qi via the spleen and kidneys [14]. TCM considers all neurodegenerative diseases such as PD, Alzheimer's disease, and motor neuron disease to be prosenescence diseases. The kidneys are believed to be the initial essence and basic power source of the body and to control the rates of growth, development, and senescence of the body [15]. If prosenescence starts at an early age, neurodegenerative diseases will develop. According to TCM theory, the kidneys have a switching function for controlling body fluids such as saliva and urine, particularly with respect to controlling the duration of opening and quantity excreted. The spleen is traditionally regarded as one source of a posteriori power since it can absorb nutrients and essence to maintain the metabolic function of the body [16]. The other important function of the spleen is to keep the fluid vessels of the body unobstructed and to keep all the body fluids circulating in the vessels (the structure may be similar to blood vessels in TCM but is too small and cannot be observed as blood vessels). If the function of the liquid circulation deteriorates due the TCM spleen (Qi deficiency), the controlling power of body fluids such as the power of saliva and urine may be weakened, and the liquid may go anywhere in irregular time. In TCM the liver is the coordinator since it can coordinate all the various functions of the viscera, including the kidneys and spleen [17]. As explained in TCM, PD can be thought of as prosenescence of the kidneys, and the causes of sialorrhea and frequent overnight urine are thought to weaken the functions of the kidneys, spleen, and liver due to Qi deficiency in the kidneys.

LSF contains eight TCM herbs.* Diaphragma juglandis *is the most important herb in TCM. It is obtained from walnuts and demonstrates a very potent effect by increasing the function (as Qi) of the kidneys (tonifying kidney function). It also contributed a potent astringency function in many clinical and basic studies which could reduce the secretion of urine and saliva [18].* Cistanche deserticola *is a well-known tonifying kidney herb in China [19–21]. It has a potent effect on the increase in the function of the kidneys and their energy source, modifies the urination time and quantity, and decreases the abnormal secretion of saliva.* Astragalus mongholicus *can increase the power of the spleen and help it to control the liquid in the liquid vessel of the body [22].* Schisandra chinensis* means literally “five-flavor berry” which is its common name. The chemical constituents of its berries include lignans schisandrin, deoxyschisandrin, gomisin, and pregomisin [23, 24], which are found in the seeds of the fruit, and it can increase the control power of the urine and saliva and together with the herbs of* Rosa laevigata *and* corn stigma *stop the secretion of abnormal fluids by the body.* Rhizoma Atractylodis *and* Radix Paeoniae Rubra *function to ensure all of the herbs complement each other and induce the eight herbs to work together to properly control the urine and saliva problems and decrease the potential adverse effects of these herbs. The study results indicate that LSF did not improve the total score of UPDRS but did improve OS, FNU, NA, and ESA, as well as the scores for ESS in the treatment group. Improvement of the frequent overnight urination was clearly demonstrated in the profile of the actigraphic recording scores (Figures 1, 2(c), and 2(d)). Sleep disturbance, which is frequent among patients with PD, is thought to be due to the disruption caused by frequent nighttime urination. Therefore, an improvement in actigraphic recording scores at night is likely a reflection of an improvement in NMS of PD after LSF treatment.

Our study has several limitations. First, the placebo granules contained five herbs, all of which have functions in TCM, although the influence is very weak. Second, the evaluation method for assessing the QA by patients or caretakers was subjective so the changes of QA were not very accurate. Third, other limitations were the study not being a RCT and the low number of cases. It left something to be desired because 6 weeks is not enough to verify the results. In order to validate the causes of the disease based on clinical data, large-scale, multicenter, double-blind randomized control studies may be needed to verify the effectiveness of LSF in the treatment of sialorrhea and frequent overnight urination in PD patients. LSF is tolerable for long-term administration and is without any intolerable adverse effects and hence is likely a suitable choice as a drug to improve the sialorrhea and frequent nighttime urination symptoms of PD.

## Figures and Tables

**Table 1:** Basal characteristics of all patients with Parkinson's disease.

	LSF (*n* = 44)	Placebo (*n* = 43)
Male/female	26/18	27/16
Age (y)	67.3 ± 9.6	66.8 ± 11.3
Age of PD onset (y)	60.5 ± 6.1	58.7 ± 7.3
Duration of PD diagnosis (y)	4.4 ± 3.3	4.7 ± 3.7
Baseline of NA (counts/min)	132.9 ± 36.5	130.3 ± 38.7
Baseline of QA (mL)	62.5 ± 36.9	62.1 ± 36.5
Baseline of FNU (times)	4.20 ± 1.7	4.18 ± 1.8
Baseline of ESA (counts/min)	166.8 ± 37.1	163.2 ± 39.4
Baseline of UPDRS	31.6 ± 6.7	31.3 ± 5.9
Baseline of ESS	9.9 ± 4.1	9.6 ± 5.2
Levodopa equivalent doses (mg)	361.6 ± 186.7	381.3 ± 175.9
Baseline of H & Y	2.5 ± 1.7	2.4 ± 1.8

*Note*. LSF: Lian-Se formula; PD: Parkinson's disease; NA: nocturnal activity; QA: quantity saliva; FNU: frequent of nighttime urination; ESA: early sleep activity; UPDRS: unified Parkinson's disease rating scale; ESS: Epworth Sleepiness Scale.
